# Macrophages enhance cisplatin resistance in gastric cancer through the transfer of circTEX2

**DOI:** 10.1111/jcmm.18070

**Published:** 2023-12-15

**Authors:** Bing Qu, Jiasheng Liu, Zhiyang Peng, Zhe Xiao, Shijun Li, Jianguo Wu, Shengbo Li, Jianfei Luo

**Affiliations:** ^1^ Department of General Surgery Renmin Hospital of Wuhan University Wuhan Hubei China

**Keywords:** circTEX2/miR‐145/ABCC1, cisplatin resistance, exosomes, gastric cancer, macrophages

## Abstract

Cisplatin‐based chemotherapy is often used in advanced gastric cancer (GC) treatment, yet resistance to cisplatin may lead to treatment failure. Mechanisms underlying cisplatin resistance remain unclear. Recent evidence highlighted the role of macrophages in cancer chemoresistance. Macrophage‐derived exosomes were shown to facilitate intercellular communication. Here, we investigated the cisplatin resistance mechanism based on macrophage‐derived exosomes in gastric cancer. Cell growth and apoptosis detection experiments revealed that M2‐polarized macrophages increased the resistance of GC cells to cisplatin. qRT‐PCR, RNAase R assay, actinomycin D assay and cell nucleo‐cytoplasmic separation experiments confirmed the existence of circTEX2 in macrophage cytoplasm, with a higher expression level in M2 macrophages than that in M1 macrophages. Further experiments showed that circTEX2 acted as microRNA sponges for miR‐145 and regulated the expression of ATP Binding Cassette Subfamily C Member 1 (ABCC1). Inhibition of the circTEX2/miR‐145/ABCC1 axis blocked the cisplatin resistance of gastric cancer induced by M2 macrophages, as evidenced by in vitro and in vivo experiments. In conclusion, our research suggests that the exosomal transfer of M2 macrophage‐derived circTEX2 enhances cisplatin resistance in gastric cancer through miR‐145/ABCC1. Additionally, communication between macrophages and cancer cells via exosomes may be a promising therapeutic target for the treatment of cisplatin‐resistant gastric cancer.

## 1 INTRODUCTION

Gastric cancer (GC) is the third most common malignancy worldwide, responsible for more than 780,000 deaths in 2019.^1^ Advanced GC patients usually survive less than a year following diagnosis.^2^ In most patients, the advanced form of GC is detected at the first diagnosis, limiting their chances of survival. Chemotherapy has been the primary treatment method for advanced GC in the past decades, with cisplatin (CDDP) being the first‐line drug. CDDP disrupts DNA repair and induces DNA damage and apoptosis in GC cells.^3^ However, CDDP resistance also leads to unsatisfactory treatment outcomes, highlighting the importance of investigating the underlying mechanism of chemoresistance in GC.

The tumour microenvironment includes various immune cell types and plays critical roles in tumour progression. Macrophages are major components of the tumour microenvironment. These cells regulate tumour progression by promoting metastasis and angiogenesis. Macrophages are also heterogeneous with two phenotypes, M1 and M2 macrophages, based on their functions in tumour progression.^4^, ^5^ In most tumours, tumour‐associated macrophages (TAMs) are primarily M2 macrophages. There is a positive correlation between M2 macrophages and poor prognosis in various cancer types,^6^, ^7^ including GC.^8^ M1 macrophages inhibit tumour progression, while M2 macrophages promote tumour progression. Inhibition of M2 macrophages and repolarization of M2 macrophages to M1 macrophages are common strategies to treat solid tumours.^9^ Thus, targeting macrophages may represent an innovative combination therapy strategy for cancer treatment.^10^ However, an in‐depth understanding of the complex interaction between cancer and macrophages is required for the development of such treatment strategies. Macrophages play important roles in modulating immune microenvironment.

Previous studies have demonstrated that cells communicate with neighbouring or distant cells by secreting exosomes, which are formed by multivesicular bodies and released into the tumour microenvironment. These exosomes contain proteins, lipids, microRNAs and, in particular, circRNAs.^11^, ^12^, ^13^ Exosomes in the tumour microenvironment can modulate the chemoresistance of cancer cells, as evidenced by previous studies.^14^, ^15^, ^16^

Circular RNAs (circRNAs) are a recently discovered class of non‐coding RNA (ncRNA), characterized by covalently closed loops and resistance to RNase R digestion.^17^, ^18^ Their unique structure renders them highly stable and they are widely present in various cell types. Advances in RNA‐sequencing technology have also led to the discovery of circRNAs in cancer cells, with key roles in cancer progression, including migration, invasion, survival, proliferation and chemoresistance.^13^, ^18^, ^19^

CircTEX2 (hsa_circ_0006479) is derived from back‐splicing of TEX2 mRNA and located on chr17:62289933‐62291602 with a length of 1669 bp. Circ‐TEX2 has a higher expression level in M2 macrophages than in M1 macrophages.^20^ However, the function of circ‐TEX2 has been unclear to date.

Here, we investigated the role of M2‐polarized macrophages in the development of cisplatin resistance in GC. Our objective was to elucidate the mechanism of communication between macrophages and cancer cells and provide a promising new therapeutic target for GC.

## 2 MATERIALS AND METHODS

### 2.1 Cell culture

Gastric cancer cells MKN45 and AGS were obtained from the Chinese Academy of Sciences Cell Bank of Type Culture Collection. Human mononuclear macrophage cell lines (THP‐1) were obtained from the American Type Culture Collection (ATCC, https://www.atcc.org/). All cells were cultured in RPMI‐1640 (Gibco; Thermo Fisher Scientific, Inc.) with 10% FBS (Invitrogen; Thermo Fisher Scientific, Inc.). All cells were cultured at 37°C with 5% CO_2_.

### 2.2 Patients and ethical approval

Gastric patients underwent primary resection at Renmin Hospital of Wuhan University (*n* = 10). A total of 10 peripheral blood samples were obtained to isolate peripheral blood mononuclear cells. This study was approved by Renmin Hospital of Wuhan University Research Ethics Committee, and patients provided their informed consent.

### 2.3 M1 and M2 macrophage polarization

Macrophages derived from THP‐1 by incubation with 200 nM phorbol 12‐myristate 13‐acetate (PMA; Sigma‐Aldrich) for 24 h. Then macrophages were with PMA plus 100 ng/mL lipopolysaccharide (LPS; Sigma) and 20 ng/mL interferon‐γ (IFN‐γ; PEPROTECH) to obtain M1 macrophages for 24 h. Then, M2‐polarized macrophages were obtained by incubation with IL‐4 (25 ng/mL; Thermo Fisher) and IL‐13 (25 ng/mL; Thermo Fisher) for another 24 h.

Lymphoprep (Fresenius Kabi‐Norge) was used to isolate peripheral blood mononuclear cells (PBMC) following the manufacturer's protocols. PBMC cells were treated with 100 ng/mL macrophage colony‐stimulating factor (M‐CSF; PEPROTECH) in an RPMI 1640 medium supplemented with 20% FBS for 6 days. To generate the M1 macrophages, the cells were treated with 100 ng/mL LPS and 20 ng/mL IFN‐γ for an additional 72 h.

### 2.4 Cell viability

Gastric cancer cells were cultured in 96 well plates at a concentration of 5000 cells per well overnight. The cells were treated with cisplatin for 48 h. Cell counting Kit‐8 (Dojindo Laboratories) was used to detect the viability of the cells following the instruction of the manufactory.

### 2.5 RNase R treatment

Total RNAs were incubated with or without 5 U/mg RNase R (Lucigen) for 20 min at 37°C. Then, RNAs were then reverse‐transcribed with Evo M‐MLV RT Premix (AccurateBiology). The level of circTEX2 and its linear mRNA was detected by realtime‐PCR.

### 2.6 Flow cytometry

M2 polarized macrophages were trypsinized and washed twice in 1× PBS, resuspended in 100 U 1× PBS, fluorochrome‐conjugated antibodies against F4/80, CD11b, CD206, CD86, CD163, CD68, CD80 or their respective isotype controls were added and stained for 30 min at 4°C. Following washed twice in 1× PBS, labelled cells were analysed by flow cytometry on a FACS Canto II flow cytometer (BD Biosciences) and analysed with FlowJo software (Tree Star).

### 2.7 Apoptosis assay

Apoptosis Detection Kit (BD) was used to detect the apoptosis of gastric cancer cells following the instructions. Gastric cancer cells were trypsinized and washed with PBS three times. Then the cells were resuspended in 100 μL binding buffer with the addition of 5 μL PI at 23°C in the dark. Fifteen minutes later, 400 μL binding buffer was added to the cells and detected by FACS Canto II flow cytometry (BD Biosciences).

### 2.8 Exosome isolation and detection

DMEM:F12 medium with 10% exosome‐free FBS was used to culture macrophages for 48 h before experiments. Exosome Precipitation Solution (System Biosciences) was used to extract exosomes from the collected medium.

### 2.9 Western blot

Gastric cancer cells were lysed in lysis buffer (Sigma–Aldrich) with protease inhibitor cocktails. Thirty microgram protein was loaded in western blot. SDS‐PAGE was used to separate the protein and then the proteins were transferred to 0.2 μm PVDF membrane (Bio‐Rad). 5% non‐fat milk was used to block the membrane. The membrane was incubated with indicated primary antibodies at the concentration of 1:1000 at 4°C overnight followed by PBS wash three times. Then, the membrane was incubated with indicated second antibodies with HPR (1:4000; LI‐COR Biosciences).

### 2.10 RNA extraction and quantitative real‐time PCR

RIzol reagent kit (Invitrogen) was used to extract the RNA from macrophages and gastric cancer cells following the instructions of the manufacturer. Nanodrop (Thermo Fisher) was used to detect the quality and quantity of RNA. PrimeScript RT mix (TaKaRa) was used for RNA reverse transcription. Mir‐X™ miRNA First‐Strand Synthesis Kit (TaKaRa) was used for microRNA expression analysis. SYBR KIT (TaKaRa) was used for quantitative real‐time PCR analysis(qRT‐PCR) with U6 or β‐actin as the endogenous control. Results were analysed using the $2^{-\Delta \Delta C_{t}}$ calculation method. The primers are shown in Table S1.

### 2.11 In vivo xenograft

Six‐week‐old male nude mice were used to establish a xenograft tumour model. 4 × 10^5^ nude mice were subcutaneously injected into the nude mice. When the tumour arrived at 5 × 5 mm, the mice with tumours were randomly divided into four groups. The mice were treated with PBS or cisplatin at the concentration of 5 mg/kg every 3 days. Exosomes from Macrophage‐NC or Macrophage‐si‐circTEX2 were intratumorally injected along with cisplatin treatment. The sizes of tumours were measured using a digital calliper every 3 days. The volumes of tumours were calculated as the following formula: volume = width^2^ × length × 0.5. All experiments on the animal were performed under the approval of Renmin Hospital of Wuhan University.

### 2.12 Statistical analysis

The significance between the two groups was calculated by a one‐way anova and a two‐tailed Student's *t* test. *p* < 0.05 was considered the difference is significant. All data were shown as means ± standard deviation (SD). All data were calculated with GraphPad Prism 8.0 (GraphPad Software). *p < 0.05, **p < 0.01, ***p < 0.001.

## 3 RESULTS

### 3.1 M2‐polarized macrophages enhance cisplatin resistance in GC cells

**FIGURE 1:**
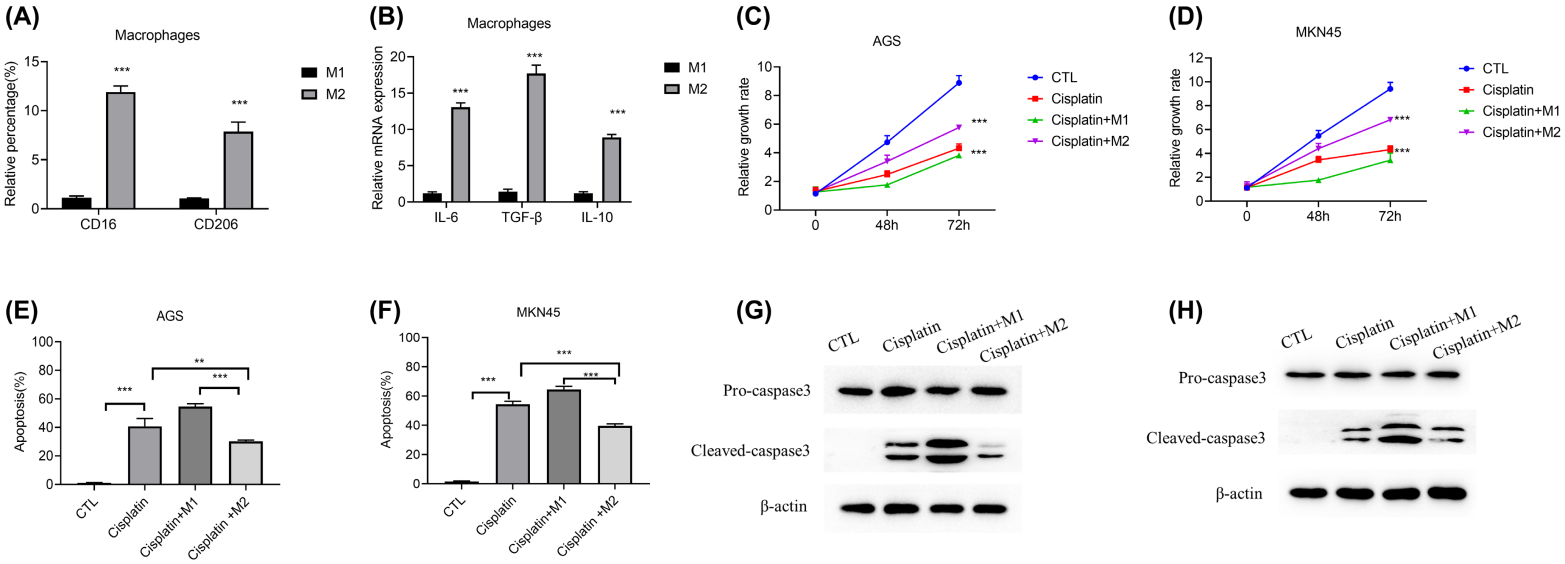
M2‐polarized macrophages enhance the cisplatin resistance of GC cells. (A) Flow cytometry was used to detect biomarkers of M2‐polarized macrophages. (B) mRNA levels of IL‐6, TGF‐β, and IL‐10. (C) Relative growth rate of AGS cells treated with cisplatin and co‐cultured with M1 or M2 macrophages. (D) Relative growth rate of MKN45 cells treated with cisplatin and co‐cultured with M1 or M2 macrophages. (E) Flow cytometry assay was used to detect apoptosis in AGS cells. AGS without cisplatin treatment was used as CTL (Control). (F) Flow cytometry assay was used to detect apoptosis in MKN45 cells. MKN45 without cisplatin treatment was used as CTL (Control). (G, H) Western blotting was performed to detect apoptosis in AGS (G) and MKN45 (H) cells. Data are presented as mean ± SD. *p < 0.05, **p < 0.01, ***p < 0.001. Each experiment was performed at least in triplicate.

We first generated M2‐polarized macrophages from THP‐1 cells to investigate their effect on cancer cells. Flow cytometry analysis was used to verify the M2‐polarized macrophage phenotype. Accordingly, the expression level of macrophage surface antigens CD16 and CD206 was found to be higher in M2‐polarized macrophages than in M1 macrophages induced by THP1 (Figure 1A). Quantitative RT‐PCR was performed to evaluate the expression of specific genes in M2 macrophages. IL‐6, TGF‐β and IL‐10 are reported to play important roles in the polarization of macrophages.^21^, ^22^, ^23^, ^24^, ^25^, ^26^, ^27^, ^28^ Expression levels of IL‐6, TGF‐β and IL‐10 were found to be increased (Figure 1B), suggesting that M2 macrophages were successfully derived from THP‐1 cells. To determine the impact of M2 macrophages on cisplatin resistance, AGS and MKN45 GC cell lines were treated with cisplatin and then co‐cultured with M1 or M2 macrophages. CCK8 assay was then used to detect the cell growth at 0, 48 and 72 h. The results showed that cisplatin effectively inhibited the growth of AGS and MKN45 cells. The strength of this growth inhibitory effect was decreased in the presence of M2 macrophages, whereas M1 macrophages enhanced the effect of cisplatin (Figure 1C,D). Flow cytometry and western blot experiments were performed to examine the effects of M2 macrophages on cisplatin‐induced apoptosis. Apoptosis experiments showed that M2 macrophages decreased cisplatin‐induced apoptosis (Figure 1E,F), which was confirmed by western blot analysis of apoptosis markers (Figure 1G,H). Therefore, these findings indicate that M2 macrophages enhance cisplatin resistance in GC cells.

### 3.2 CircTEX2 is upregulated in M2 macrophages

**FIGURE 2:**
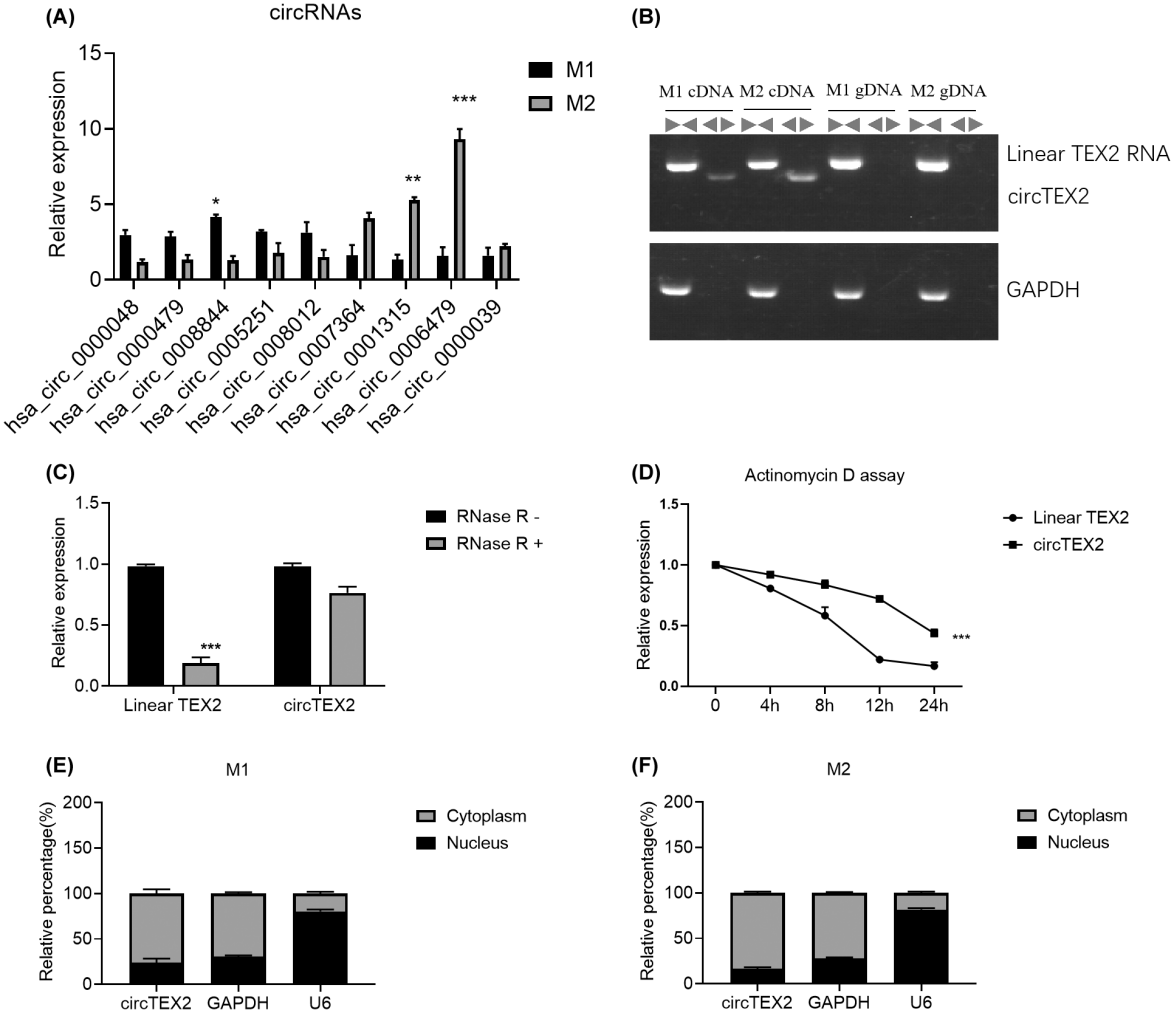
CircTEX2 is upregulated in M2 macrophages. (A) qRT‐PCR was used to quantify circRNA expression levels in M1 and M2 macrophages. (B) Convergent and divergent primers designed specifically were used to detect circTEX2 and linear isoform expressions in cDNA and gDNA of M1 or M2 macrophages. (C) RNase R assay was performed on M2 macrophages. (D) Actinomycin D assay was performed on M2 macrophages. (E‐F) The location of circTEX2 was detected in M1 and M2 macrophages. Data are presented as mean ± SD. *p < 0.05, **p < 0.01, ***p < 0.001. Each experiment was performed at least in triplicate.

circRNAs have been shown to play a significant role in macrophage polarization.^29^, ^30^, ^31^ We investigated the role of circRNA in macrophage polarization using qRT‐PCR to detect the differences in circRNA between M1 and M2 macrophages. Our results revealed a significant upregulation of circTEX2 (hsa_circ_0006479) in M2 macrophages (Figure 2A). We designed convergent and divergent primers and extracted gDNA (genomic DNA) and cDNA (complementary DNA) from AGS and MKN45 cells to confirm the unique structure of circTEX2. PCR and agarose gel electrophoresis analysis results also indicated that circTEX2 was only detected in cDNA by divergent primers. These results confirmed the unique head‐to‐tail splicing loop structure of circTEX2. RNase R assay results revealed that circTEX2 was more resistant to RNase R digestion than the linear isoform (Figure 2C). Additionally, we found that circTEX2 was more stable than linear TEX2 when treated with a transcription inhibitor, actinomycin D. We also determined the subcellular localisation of circTEX2 in macrophages since localisation is closely related to the function of circRNAs. Our results showed that circTEX2 was mainly found in the cytoplasm of both M1 and M2 macrophages (Figure 2E,F). These results suggest that circTEX2 is upregulated in M2 macrophages and may act as a microRNA sponge.

### 3.3 Exosomes derived from M2‐polarized macrophages enhance cisplatin resistance in GC cells via circTEX2

**FIGURE 3:**
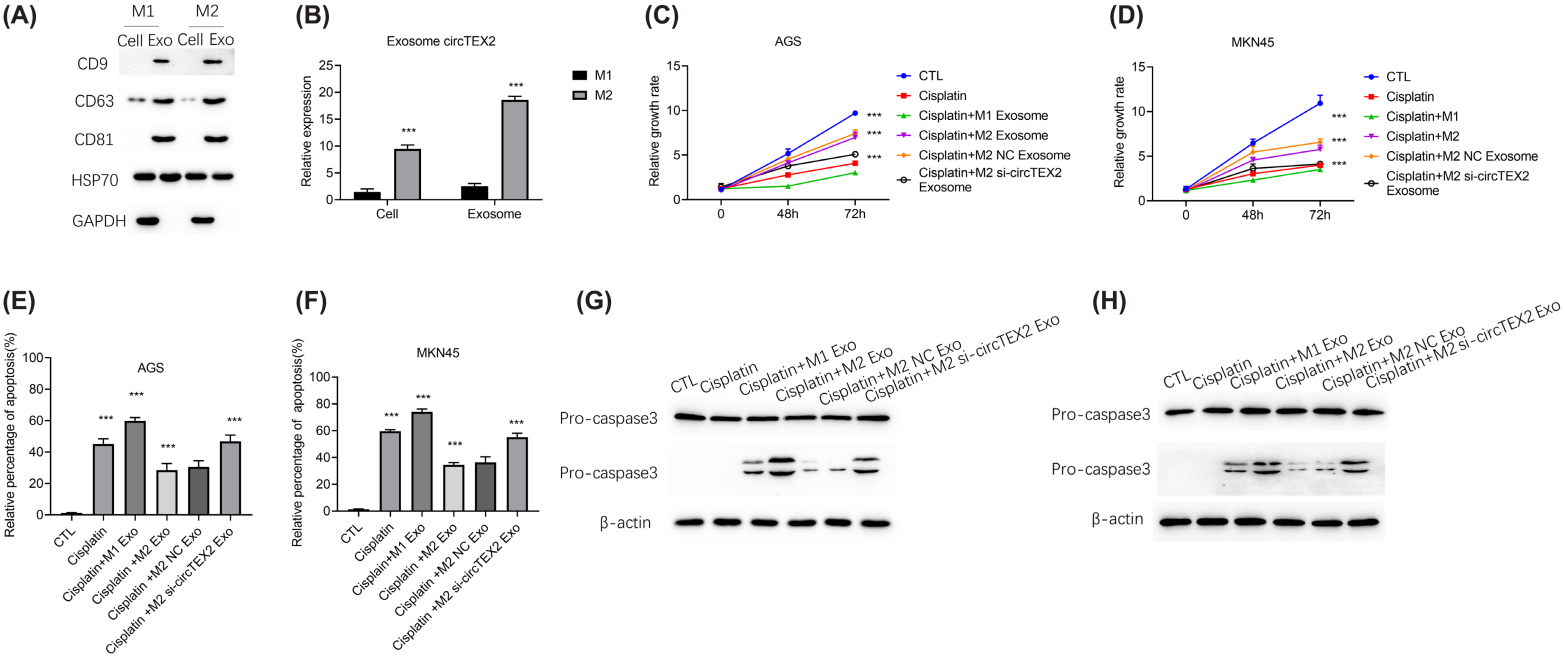
Exosomes derived from M2‐polarized macrophages enhance cisplatin resistance in GC cells via circTEX2. (A) Western blotting was used to verify the extraction of macrophage‐derived exosomes. (B) qRT‐PCR was used to determine the expression level of circTEX2 in macrophages and derived exosomes. (C, D) CCK8 assay was used to detect growth rates of AGS (C) and MKN45 (D) cells with the indicated treatment. CTL is short for control group. (E, F) Flow cytometry assay was used to detect apoptosis in AGS (E) and MKN45 (F) cells with indicated treatment. (G, H) Western blotting was used to detect apoptosis in AGS (G) and MKN45 (H) cells with indicated treatment. Data are presented as mean ± SD. *p < 0.05, **p < 0.01, ***p < 0.001. Each experiment was performed at least in triplicate.

Macrophages can communicate with neighbouring or distant cells by secreting exosomes into their environment.^32^, ^33^, ^34^ We isolated exosomes from the M2 macrophage‐conditioned medium (CM) to investigate whether M2 macrophages play a role in chemoresistance through exosome secretion. Western blotting was used to detect exosomal markers CD9, CD63 and CD81 (Figure 3A), whereas qRT‐PCR was used to detect the level of circTEX2 in the exosomes. The results showed that circTEX2 was enriched in exosomes (Figure 3B). Treatment with M2‐derived exosomes reduced cisplatin sensitivity and improved the survival of AGS cells, as evidenced by the results of CCK8 assays (Figure 3C). Furthermore, AGS cells co‐cultured with M2 macrophage‐derived exosomes exhibited a lower level of apoptosis than cells co‐cultured with M1 macrophage‐derived exosomes (Figure 3E,G). Silencing of circTEX2 abrogated the effect of exosomes derived from M2 macrophages (Figure 3C,E,G). Similar results were obtained in MKN45 cells (Figure 3D,F,H). Collectively, these findings indicate that M2 macrophage‐derived exosomes enhance GC cisplatin resistance through circTEX2.

### 3.4 CircTEX2 functions as a microRNA sponge for miR‐145

**FIGURE 4:**
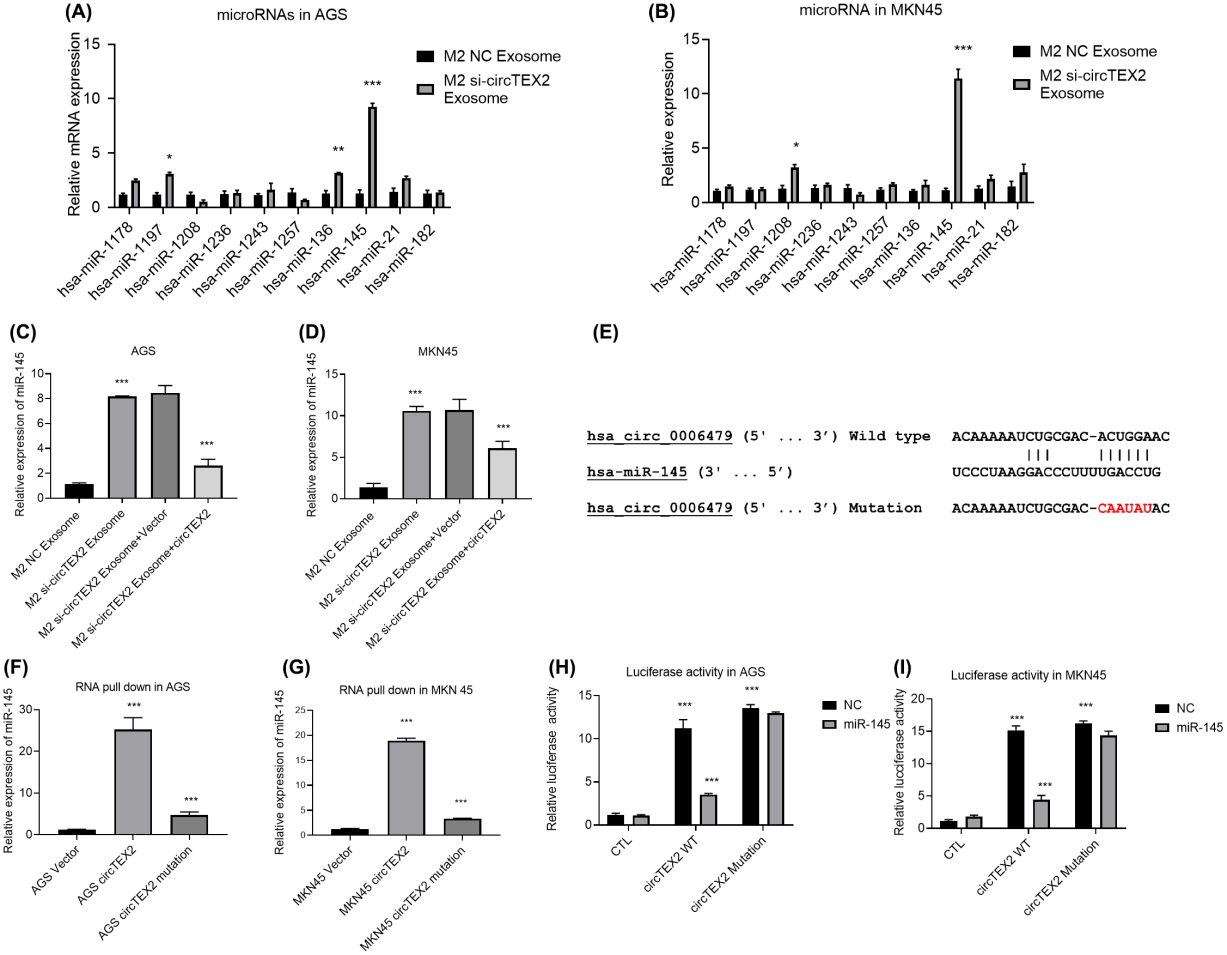
CircTEX2 acts as a microRNA sponge for miR‐145. (A) qRT‐PCR was used to detect microRNA levels in AGS cells with the treatment of M2 NC exosomes or si‐circTEX2 exosomes. NC is short for nonsense control. (B) qRT‐PCR was used to quantify microRNA levels in MKN45 cells with the treatment of M2 NC exosomes or si‐circTEX2 exosomes. (C) qRT‐PCR was used to detect the level of miR‐145 in AGS (C) and MKN45 (D) cells. (E) Mutation of the predicted binding site on circTEX2. (F) RNA pull‐down assay was performed on AGS cells. (G) RNA pull‐down was performed on MKN45 cells. (H) Luciferase activity assay was performed on AGS cells. (I) Luciferase activity assay was performed on MKN45 cells. Data are presented as mean ± SD. *p < 0.05, **p < 0.01, ***p < 0.001. Each experiment was performed at least in triplicate.

Previous studies have demonstrated that circRNAs act as microRNA sponges to regulate downstream target gene expression in tumour development.^35^, ^36^, ^37^ We used the Circular RNA Interactome (CircInteractome, https://circinteractome.irp.nia.nih.gov/) to identify potential target microRNAs for circTEX2. We selected 10 candidate microRNAs based on the results of our prediction and previous studies and performed qRT‐PCR to detect their expression of AGS and MKN45 co‐cultured with M2 macrophages NC or si‐circTEX2‐derived exosomes. Our results showed that exosomes from M2‐si‐circTEX2 macrophages significantly increased the expression of miR‐145 in both AGS (Figure 4A) and MKN45 cells (Figure 4B). To confirm the regulatory role of circTEX2, we re‐expressed circTEX2 in GC cells and found that the re‐expression of circTEX2 blocked the upregulation of miR‐145, which was induced by silencing of circTEX2 (Figure 4C,D). To investigate the presence of a direct interaction between miR1‐45 and circTEX2, we mutated the predicted binding sites (Figure 4E) and performed RNA pull‐down (Figure 4F,G) and luciferase activity assays (Figure 4H,I) in GC cells. Our results showed that biotin‐labelled circTEX2 exhibited significant enrichment of miR‐145 compared with the control group. This enrichment effect disappeared upon mutation of the binding site (Figure 4F,G). Luciferase activity assays also showed that miR‐145 significantly affected the luciferase activity of wild‐type circTEX2 without influencing the activity of the binding site‐mutated circTEX2 (Figure 4H,I). These results demonstrated that circTEX2 can act as a microRNA sponge for miR‐145.

### 3.5 CircTEX2/miR‐145 regulates the expression of ABCC1

**FIGURE 5:**
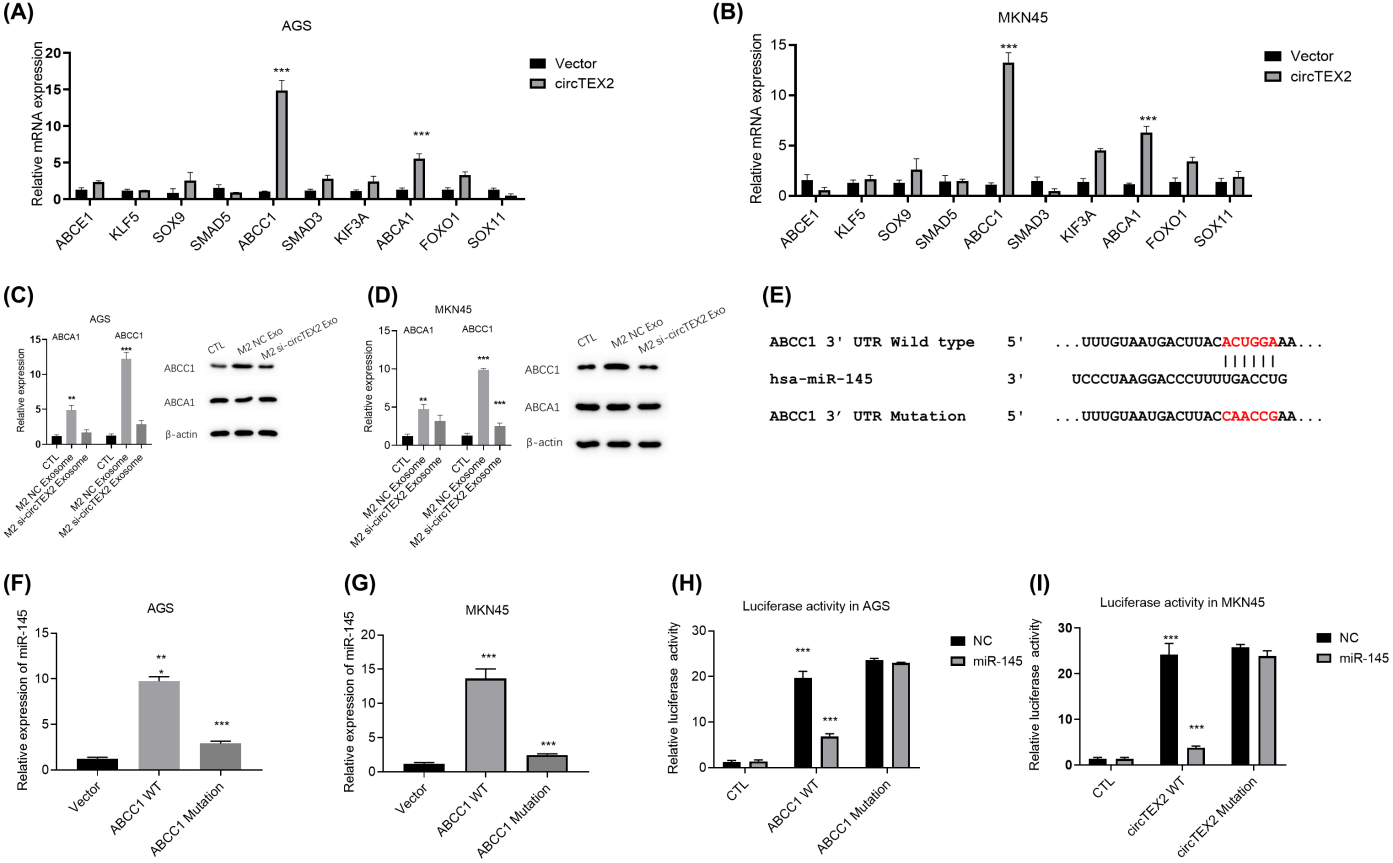
CircTEX2/miR‐145 regulates the expression of ABCC1. (A, B) qRT‐PCR assay was used to quantify the expression of predicted target genes in AGS (A) and MKN45 (B) cells. (C, D) qRT‐PCR and western blotting were used to determine the mRNA and protein levels of ABCC1 and ABCA1 in AGS (C) and MKN45 (D) cells. (E) Mutation of the predicted binding site on ABCC1 3′UTR. (F) RNA pull‐down assay was performed on AGS cells. (G) RNA pull‐down assay was performed on MKN45 cells. (H) Luciferase activity assay was performed on AGS cells. (I) Luciferase activity assay was performed on MKN45 cells. Data are presented as mean ± SD. *p < 0.05, **p < 0.01, ***p < 0.001. Each experiment was performed at least in triplicate.

We performed further experiments to identify the downstream targets of circTEX2/miR‐145 by performing a comparative evaluation of miRDB and Targetscan databases. We detected 10 possible genes based on prediction results and findings from previous studies. qRT‐PCR results showed upregulation of *ABCC1* in both AGS (Figure 5A) and MKN45 (Figure 5B) cells following transfection with circTEX2, suggesting that circTEX2 upregulates *ABCC1* and *ABCA1*. To confirm the downstream regulation of circTEX2, we further cultured AGS and MKN45 with M2 NC and M2 si‐circTEX2 exosomes. Then, we performed qRT‐PCR and western blotting analyses to detect the expression of *ABCC1* and *ABCA1*. Accordingly, M2 NC significantly upregulated the mRNA and protein levels of *ABCC1* (Figure 5C), whereas the M2 si‐circTEX2 exosome did not affect the expression of ABCC1. However, neither M2 NC nor M2 si‐circTEX2 exosomes affected the protein levels of *ABCA1* in AGS cells (Figure 5C). Similar results were obtained in MKN45 cells (Figure 5D). To investigate whether circTEX2 regulates the expression of *ABCC1* through miR‐145, we performed RNA pull‐down and luciferase activity assays with wild‐type and mutated *ABCC1* 3′UTR. We mutated the predicted binding site, as shown in Figure 5E. RNA pull‐down assay showed that wild‐type *ABCC1* 3′UTR enhanced the expression of miR‐145 compared with the vector group (Figure 5F,G). *ABCC1* 3′UTR with mutation did not show the same effect (Figure 5F,G). Transfection of miR‐145 decreased the luciferase activity of wild‐type *ABCC1* 3′UTR and did not significantly affect the luciferase activity of the mutated *ABCC1* 3′UTR (Figure 5H,I). These results confirmed *ABCC1* as the downstream target gene of miR‐145.

### 3.6 Blocking the circTEX2/miR‐145/ABCC1 axis enhanced cisplatin sensitivity in GC

**FIGURE 6:**
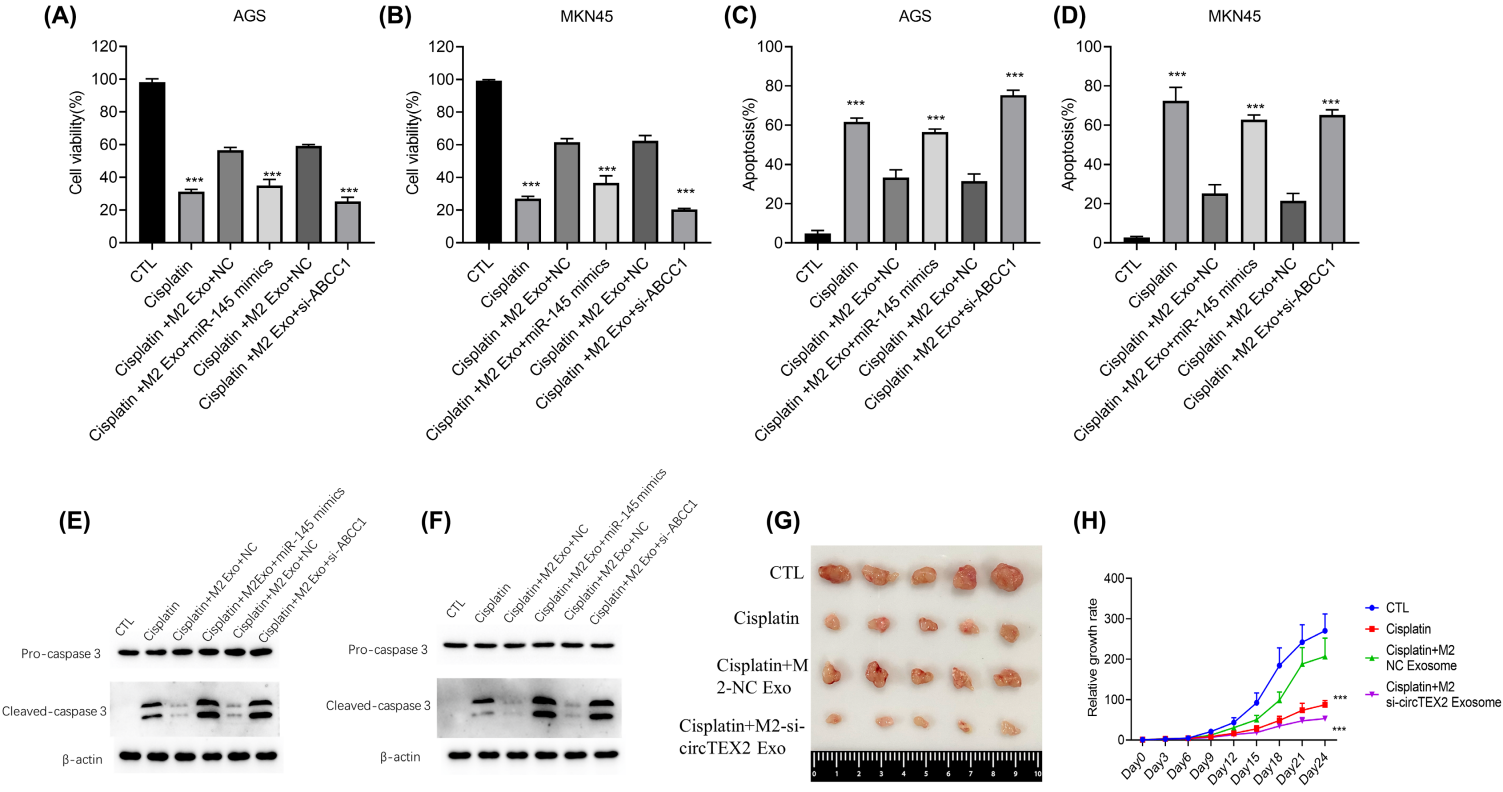
Blocking of the circTEX2/miR‐145/ABCC1 axis enhances the cisplatin sensitivity of gastric cancer cells. (A, B) CCK8 assay was used to determine cell viability in AGS (A) and MKN45 (B) cells with the cisplatin treatment. (C, D) Apoptosis was detected by cytometry in AGS (C) and MKN45 (D) cells treated with cisplatin. (E, F) Apoptosis was detected by western blotting in AGS (E) and MKN45 (F) cells with the cisplatin treatment. (G, H) The sizes and growth rates of tumours from nude mice inoculated with AGS cells with treatment of cisplatin and macrophage‐derived exosomes (*n* = 5). Data are presented as mean ± SD. *p < 0.05, **p < 0.01, ***p < 0.001. Each experiment was performed at least in triplicate.

We further performed cell viability and apoptosis assays to investigate the effect of circTEX2/miR‐145/ABCC1 on the cisplatin sensitivity of GC cells. CCK8 assay showed that transfections of miR‐145 mimics or *ABCC1* siRNAs in GC cells decreased cell viability compared with the control group with M2‐derived exosomes in both AGS (Figure 6A) and MKN45 cells (Figure 6B). Apoptosis assay showed that transfection of miR‐145 mimics or *ABCC1* siRNAs significantly increased the cisplatin‐induced apoptosis in GC cells compared with the control group co‐cultured with M2‐derived exosomes (Figure 6C,D). These results were also confirmed by the detection of the apoptosis protein marker caspase3 by western blotting (Figure 6E,F). To investigate the role of M2 macrophage‐derived exosomes in chemoresistance in vivo, AGS cells were subcutaneously injected into nude mice. When the tumour size reached 5 × 5 mm, the nude mice were randomly divided into four groups. The tumours were treated with M2‐NC exosomes or M2‐si‐circTEX2 exosomes followed by cisplatin and their characteristics were compared with those of the control groups. We found that M2‐NC exosomes significantly enhanced cisplatin resistance in AGS, whereas M2‐si‐circTEX2 exosome did not significantly affect the cisplatin resistance (Figure 6G,H). Taken together, these results suggested that M2 macrophage‐derived exosomes induce cisplatin resistance in GC cells through circTEX2 both in vitro and in vivo.

### 3.7 circTEX2 contributed to human monocyte‐derived M2 macrophages‐related cisplatin resistance

**FIGURE 7:**
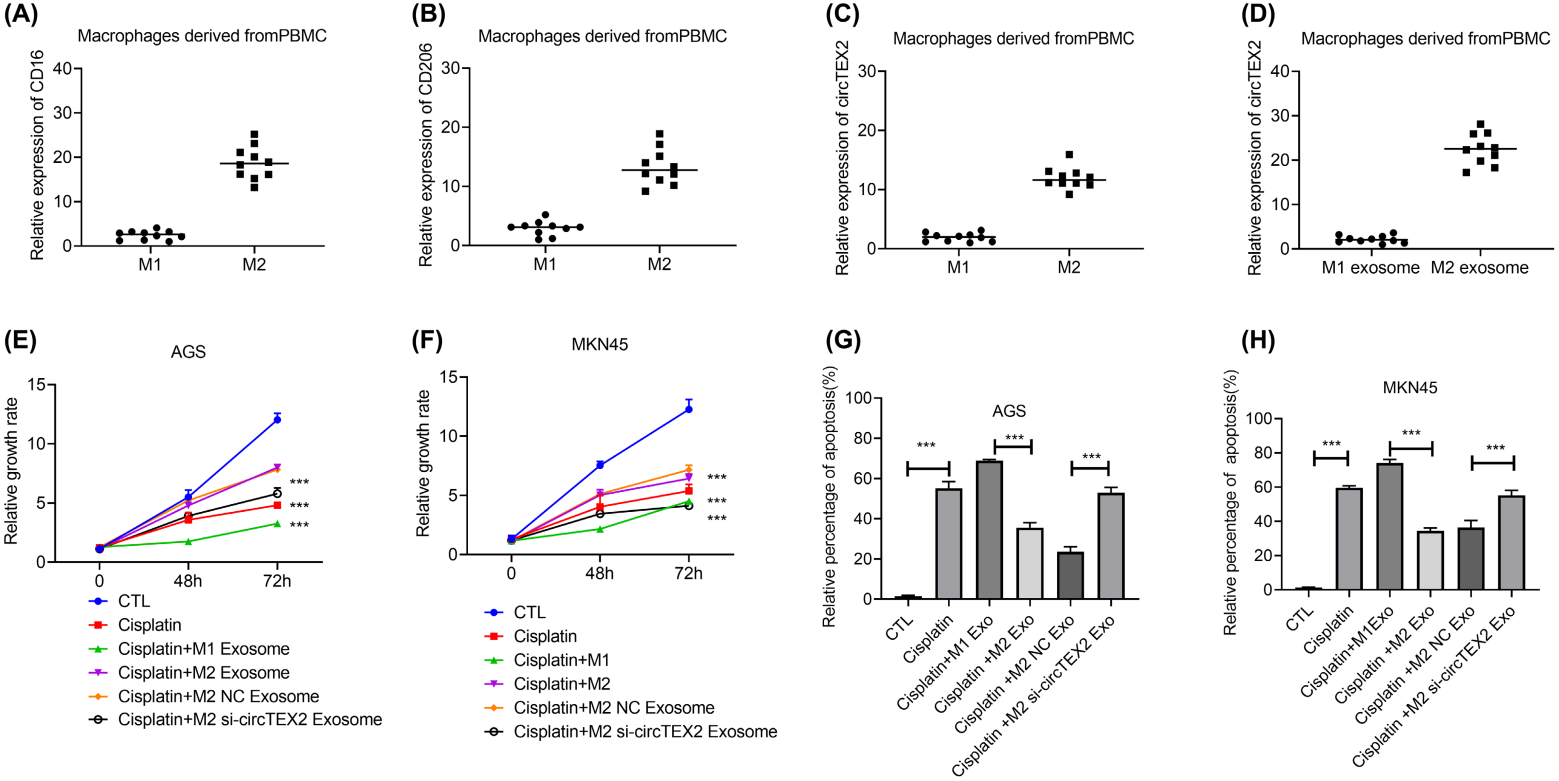
The function of circTEX2 in human monocyte‐derived macrophages induced cisplatin resistance. (A) The mRNA levels of CD16 (A) and CD206 (B) in human monocyte‐derived M1 and M2 macrophages. (C) The level of circTEX2 in human monocyte‐derived M1 and M2 macrophages. (D) The level of circTEX2 in exosomes of human monocyte‐derived M2 macrophages. (E, F) CCK8 assays were used to detect the cell growth with the indicated treatment. (G, H) Apoptosis assays were used to detect the cell growth with indicated treatment. Data are presented as mean ± SD. *p < 0.05, **p < 0.01, ***p < 0.001. Each experiment was performed at least in triplicate.

To confirm the function of circTEX2 in human monocyte‐derived macrophages, we collected and extracted human peripheral blood mononuclear cells from gastric cancer patients. The macrophages were induced into M1 and M2 macrophages (Figure 7A,B). qPCR results showed that circTEX2 had higher expressions in human monocyte‐derived M2 macrophages (Figure 7C). The expressions of circTEX2 were also higher in exosomes of human monocyte‐derived M2 macrophages which were in accordance with in vitro experiments (Figure 7D). CCK8 and apoptosis assays showed that macrophage M2 and M2‐derived exosomes enhanced the resistance to cisplatin, while silence of circTEX2 abrogated the function of M2 exosomes (Figure 7E–H).

## 4 DISCUSSION

Cisplatin‐based chemotherapy is widely used to treat GC. However, its clinical use is limited due to the development of cisplatin resistance in advanced GC patients.^38^ The tumour microenvironment, specifically macrophages, was previously reported to play critical roles in tumour progression, growth, invasion, metastasis, angiogenesis and drug resistance by releasing various types of factors.^39^ Here, we found that M2 macrophages enhanced GC resistance to cisplatin by releasing exosomes that included higher levels of circTEX2 than those released by M1 macrophages. The transfer of circTEX2 from M2 macrophages to GC cells promoted cisplatin resistance by downregulating miR‐145, which further regulated the downstream target gene *ABCC1*. Our study thus demonstrated that M2 macrophages enhance cisplatin resistance of GC cells through the exosomal transfer of circTEX2, which regulates the miR‐145/ABCC1 pathway.

Exosomes serve as crucial mediators in cell‐to‐cell communication within the tumour microenvironment. Previous studies have shown that the delivery of exosomal cargo‐containing DNA, RNA and proteins was associated with chemoresistance in cancer.^39^, ^40^, ^41^ Ba demonstrated that exosome‐delivered circRNA promotes glycolysis to induce chemoresistance through the miR‐122‐PKM2 axis in colorectal cancer. Yayi Hou suggested that carcinoma‐associated fibroblasts promote the stemness and chemoresistance of colorectal cancer by transferring exosomal lncRNA H19.^42^ Similarly, we demonstrated that M2 macrophage‐derived exosomes are sufficient to enhance cisplatin resistance in GC both in vivo and in vitro, suggesting that M2 macrophages may contribute to chemoresistance in GC by releasing exosomes without physical contact in the tumour microenvironment.

circRNAs have been shown to play critical roles in GC drug resistance in recent years.^42^, ^43^ circRNAs are mainly located in the cytoplasm and serve as ‘microRNA sponges’ that protect the target gene from microRNA degradation.^44^ For example, circSHKBP1 sponges miR‐582‐3p to increase HUR expression, thereby promoting GC progression.^45^ Likewise, circNRIP1 sponges miR‐149‐5p to regulate *AKT1* expression.^46^ Moreover, the exosome‐mediated transfer of circRNA in the tumour microenvironment is increasingly recognized to play a critical role in chemoresistance in cancer.^16^, ^47^

The cell nucleo‐cytoplasmic separation experiment confirmed that circTEX2 is mainly found in the cytoplasm. qRT‐PCR and RNA‐pull‐down assays also showed that circTEX2 binds to miR‐145. Furthermore, the luciferase reporter assay confirmed the presence of a direct interaction between circTEX2 and miR‐145. Mutations in the binding site were found to abrogate the interaction between circTEX2 and miR‐145 (Figure 4).

Next, we aimed to determine the biological function of miR‐145 and its downstream target genes. For this purpose, we searched for the target genes of miR‐145 by cross‐referencing miRDB and Targetscan databases. qRT‐PCR and western blot were used to confirm the effect of miR‐145 on *ABCC1* RNA and protein levels respectively. RNA pull‐down and luciferase activity assays also confirmed the direct interaction between miR‐145 and ABCC1 3′UTR and identified the binding site (Figure 5).

The multidrug resistance protein 1 (MRP1) encoded by ABCC1 was initially identified as a factor of cancer multidrug resistance. The ABC transporter family is the largest transporter superfamily known to date,^48^ with a total of 48 transporters. The ABC transporter family is divided into seven subgroups based on their nucleotide and protein sequences (ABCA‐ABCG).^49^ ABCB1/P‐gp (P‐glycoprotein), multidrug resistance‐associated protein 1 (ABCC1/MRP1) and breast cancer resistance protein (ABCG2/BCRP) are the most studied ABC transporters with well‐established roles in mediating multidrug resistance in cancer cell models.^50^, ^51^, ^52^ ABCC1, the first transporter to be identified and characterized as a contributor to multidrug chemoresistance in human small‐cell lung carcinoma cell lines,^53^ is reported to be closely involved in multidrug resistance development in various cancers.^54^ An extraordinarily wide variety of chemotherapeutic drugs have been reported as substrates of ABCC1, such as etoposide, cisplatin, paclitaxel and vincristine.^55^ We found that M2 macrophages transfer circTEX2 to cancer cells by secreting exosomes. CircTEX2 regulates the expression of ABCC1 by sponging miR‐145. Inhibition of ABCC1 blocked circTEX2/miR‐145 induced cisplatin resistance in GC both in vitro and in vivo. Our findings suggest a new pathway of ABCC1 regulation, providing new insights into chemoresistance induced by ABCC1 overexpression. Our findings also indicate that ABCC1 regulation may be a viable approach to improve the effectiveness of chemotherapy in cancer treatment.

In conclusion, we found here that M2 macrophages contribute to cisplatin resistance in GC through the transfer of exosomal circTEX2. Our findings suggest that circTEX2 acts as a sponge for miR‐145 and regulates the expression of ABCC1. Inhibition of the miR‐145/ABCC1 axis abrogates circTEX2‐induced cisplatin resistance. Targeting exosomal circTEX2 holds promise as a therapeutic strategy for GC.

## AUTHOR CONTRIBUTIONS

**Bing Qu:** Project administration (lead); resources (equal); writing – original draft (equal). **Jiasheng Liu:** Methodology (equal); software (equal). **Zhiyang Peng:** Investigation (equal); validation (equal). **Zhe Xiao:** Formal analysis (equal); supervision (equal). **Shijun Li:** Data curation (equal); supervision (equal); visualization (equal). **Jianguo Wu:** Conceptualization (equal); visualization (equal). **Shengbo Li:** Data curation (equal); formal analysis (equal). **Jianfei Luo:** Funding acquisition (equal); resources (equal); supervision (equal); writing – original draft (equal).

## FUNDING INFORMATION

This work was supported by the Fundamental Research Funds for the Central Universities (No. 2042023kf0223); and the Interdisciplinary Innovative Talents Foundation from Renmin Hospital of Wuhan University (JCRCWL‐2022‐006); the Scientific Research Foundation of Hubei Microcirculation Society (No. HBWXH20220101) and the Natural Science Foundation of Hubei Province (2013CFB272).

## CONFLICT OF INTEREST STATEMENT

The authors confirmed that there is no conflict of interest.

## Supporting information


Table S1.

## Data Availability

The data of this study are available from the corresponding author upon reasonable request.
