# Supplementary material for: Macrophages enhance cisplatin resistance in gastric cancer through the transfer of circTEX2
Source: J Cell Mol Med. 2023 Dec 15;28(5):e18070. doi: 10.1111/jcmm.18070 (PMC10902310; doi:10.1111/jcmm.18070)
Supplement: Supplementary file 1 — Table S1. [file JCMM-28-e18070-s001.docx]

**Supplementary table: Primers for quantitative real-time PCR**

| **Gene** | **Forward Primer** | **Reverse Primer** |
| --- | --- | --- |
| ABCE1 | GGAATGCAAAAAGAGTTGTCCTG | CGAGGGATAGGCAACCTGTG |
| KLF5 | CCTGGTCCAGACAAGATGTGA | GAACTGGTCTACGACTGAGGC |
| SOX9 | AGCGAACGCACATCAAGAC | CTGTAGGCGATCTGTTGGGG |
| SMAD5 | CCAGCAGTAAAGCGATTGTTGG | GGGGTAAGCCTTTTCTGTGAG |
| ABCC1 | GTGAATCGTGGCATCGACATA | GCTTGGGACGGAAGGGAATC |
| SMAD3 | TGGACGCAGGTTCTCCAAAC | CCGGCTCGCAGTAGGTAAC |
| KIF3A | GTGTTCGAGCTATTCCTGAACTT | CCTCTAACCTTTGTGTCTGATCC |
| ABCA1 | GGAAGAACAGTCATTGGGACAC | GCTACAAACCCTTTTAGCCAGT |
| FOXO1 | TCGTCATAATCTGTCCCTACACA | CGGCTTCGGCTCTTAGCAAA |
| SOX11 | AGCAAGAAATGCGGCAAGC | ATCCAGAAACACGCACTTGAC |
| β-actin | CATGTACGTTGCTATCCAGGC | CTCCTTAATGTCACGCACGAT |
